# Exploring trust factors in AI-healthcare integration: a rapid review

**DOI:** 10.3389/frai.2025.1658510

**Published:** 2025-12-04

**Authors:** Megan Mertz, Kelvi Toskovich, Gavin Shields, Ghislaine Attema, Jennifer Dumond, Erin Cameron

**Affiliations:** 1 Dr. Gilles Arcand Centre for Health Equity, NOSM University, Thunder Bay, ON, Canada; 2 Faculty of Education, Lakehead University, Thunder Bay, ON, Canada; 3 Health Sciences Library, NOSM University, Thunder Bay, ON, Canada; 4 Human Sciences Division, NOSM University, Thunder Bay, ON, Canada

**Keywords:** artificial intelligence, trust, trust factors, healthcare, AI, factors impacting trust, AI integration

## Abstract

This rapid review explores how artificial intelligence (AI) is integrated into healthcare and examines the factors influencing trust between users and AI systems. By systematically identifying trust-related determinants, this review provides actionable insights to support effective AI adoption in clinical settings. A comprehensive search of MEDLINE (Ovid), Embase (Ovid), and CINAHL (Ebsco) using keywords related to AI, healthcare, and trust yielded 872 unique citations, of which 40 studies met the inclusion criteria after screening. Three core themes were identified. AI literacy highlights the importance of user understanding of AI inputs, processes, and outputs in fostering trust among patients and clinicians. AI psychology reflects demographic and experiential influences on trust, such as age, gender, and prior AI exposure. AI utility emphasizes perceived usefulness, system efficiency, and integration within clinical workflows. Additional considerations include anthropomorphism, privacy and security concerns, and trust-repair mechanisms following system errors, particularly in high-risk clinical contexts. Overall, this review advances the understanding of trustworthy AI in healthcare and offers guidance for future implementation strategies and policy development.

## Introduction

Artificial intelligence (AI) refers to software systems capable of performing tasks that mimic human reasoning through data processing and algorithms (Kaplan et al., 2023; West, 2018). As open-source AI platforms have expanded, adoption across sectors—including healthcare—has accelerated, heightening the need to understand and establish trust in these technologies (Lyons et al., 2024). When appropriately applied, AI can enhance task accuracy, improve safety, and support decision-making efficiency (Baduge et al., 2022; Sardar et al., 2019; Soori et al., 2023; Abdar et al., 2022; Begoli et al., 2019). In healthcare, AI is increasingly used to streamline workflows and reduce human error. Examples include electronic medical record (EMR) automation to support coordinated care (Rojahn et al., 2023), clinical decision support systems (CDSS) that aid diagnosis and treatment planning (Laxar et al., 2023; Osheroff et al., 2007), and robot-assisted surgery designed to minimize procedural risks (Hamet and Tremblay, 2017; Soori et al., 2023). Despite these benefits, challenges remain, such as inaccurate chatbot responses, diagnostic errors, and risks of exacerbating health inequities (Kaplan et al., 2023; D’Elia et al., 2022). Growing concerns around reliability, bias, transparency, and ethical use make trust a critical component of successful AI integration (Kostick-Quenet et al., 2024; Asan et al., 2020).

Trust involves vulnerability and uncertainty and shapes how individuals engage with automated systems as much as with other people (Deng et al., 2024; Hoff and Bashir, 2015). Both insufficient trust—leading to system avoidance—and overtrust—leading to misuse—pose risks (Muir and Moray, 1996). In healthcare, trust underpins patient-provider relationships and clinician reliance on diagnostic and treatment supports (Birkhäuer et al., 2017; Gaube et al., 2021). Trust has long been fundamental to healthcare, shaping both patient–clinician relationships and clinical decision-making processes. Patients must trust that clinicians act in their best interests and possess the expertise to deliver safe, effective care (Birkhäuer et al., 2017). Likewise, clinicians must have confidence in the tools and systems that support their diagnostic and treatment decisions (Gaube et al., 2021). As AI becomes increasingly embedded in clinical workflows, establishing trust from both patients and clinicians is essential for adoption and meaningful use (Asan et al., 2020). Ultimately, trust determines whether AI technologies are accepted, integrated into practice, and relied upon in patient care, underscoring the need to understand the evolving dynamics of human-machine trust in healthcare (Asan et al., 2020; Gaube et al., 2021; Lee and See, 2004). Therefore, fostering trust in AI requires addressing the needs and expectations of both clinicians and patients (Asan et al., 2020).

This rapid review synthesizes current evidence on the factors shaping human-machine trust in healthcare, with the aim of informing safe, effective, and trusted AI implementation.

## Methods

The search strategy for this rapid review was developed in consultation with a Health Sciences Librarian. Searches were conducted on March 22, 2024, in MEDLINE (Ovid), Embase (Ovid), and CINAHL (Ebsco). These databases were chosen for this rapid review due to their extensive coverage and relevance to medical and allied health literature, especially in relation to the application of artificial intelligence in healthcare.

To capture the scope of the published research, the final search strategy relies on Medical Subject Headings (MeSH) from MEDLINE (Ovid) and is translated to the equivalent term in the other resources. Target articles were also reviewed for relevant subject headings. Previous iterations of the search strategy were complex, and many citations were found to be too specific for this review. Because of the complex and individualized nature of trust relationships, we decided not to define trust before commencing our study (Table 1).

**Table 1:** A summary of the comprehensive database search strategy used, including search terms, Boolean operators, and databases (MEDLINE, Embase, and CINAHL) employed to identify studies.

Key terms	Subject headings
*Artificial Intelligence*	Artificial Intelligence/ (MEDLINE) Artificial Intelligence/ (Embase) MH “Artificial Intelligence+” (CINAHL)
*Expert Systems*	Expert Systems/ (MEDLINE) Expert System/ (Embase) MH “Expert Systems” (CINAHL)
*Knowledge Bases*	Knowledge Bases/ (MEDLINE) Knowledge Base/ (Embase) MH “Knowledge Bases+” (CINAHL)
*Machine Learning*	Exp Machine Learning/ (MEDLINE) Machine Learning/ (Embase) MH “Machine Learning+” (CINAHL)
*Natural Language Processing*	Natural Language Processing/ (MEDLINE) Natural Language Processing/ (Embase) MH “Natural Language Processing” (CINAHL)
*Neural Networks, Computers*	Neural Networks, Computers/ (MEDLINE) Exp Artificial Neural Network/ (Embase) MH “Neural Networks (Computer)” (CINAHL)
*Robotics*	Robotics/ (MEDLINE) Robotics/ (Embase) MH “Robotics” (CINAHL)
*Trust*	Trust/ (MEDLINE) Trust/ (Embase) MH “Trust” (CINAHL)
*Delivery of Health Care*	Exp “Delivery of Health Care”

The forward slash (/) denotes a medical subject heading in MEDLINE and Embase. Exp denotes the exploding of a medical subject heading in MEDLINE & Embase. MH denotes exact subject heading in CINAHL. + denotes the exploding of an exact subject heading in CINAHL.

## Results

### Search results

**Figure 1:**
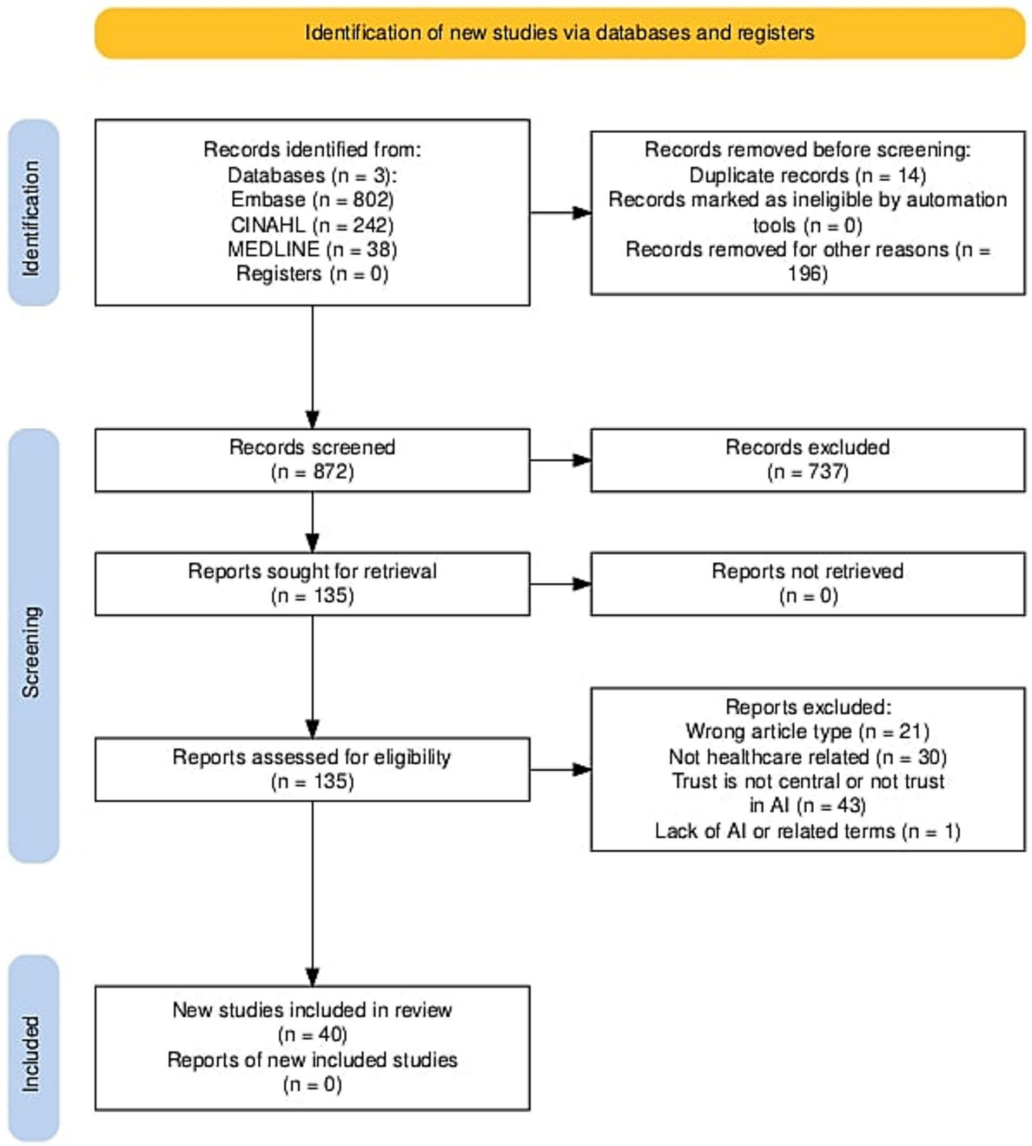
PRISMA diagram of search results and article selection.

Figure 1 summarizes the search results and article selection process. A search of three databases identified 1,082 (EMBASE *n* = 802; CINAHL *n* = 242; MEDLINE *n* = 38) articles for screening. All duplicates and editorial articles were removed (*n* = 210), and a total of 872 articles underwent title, abstract and citation screening. This process excluded 737 articles, leaving 135 articles for full-text screening. 135 full-text articles were assessed for eligibility, with a further 95 articles being excluded because of the wrong article type (*n* = 21), not related to healthcare (*n* = 30), trust was not a central aspect of the article/the paper did not relate to trust in AI (*n* = 43), and a lack of AI or related terms (*n* = 1). Data was extracted and analyzed from the remaining 40 articles.

### Study characteristics

Of the 40 included studies, 20 were quantitative, 12 were qualitative, and 8 were mixed methods (Table 2). A summary of the specific healthcare domains is included in Table 2, although many (*n* = 11) took a broad approach and did not identify a specific area of healthcare.

**Table 2:** Summary of included studies examining trust in healthcare AI systems.

Title	Author(s) (Year)	Journal	Primary Author Affiliation (Country)	Type of Artificial Intelligence	Primary Factors of Trust	Domain (education, service delivery, research)
Elicitation of trustworthiness requirements for highly dexterous teleoperation systems with signal latency	Louca et al. (2023)	Frontiers in Neurorobotics	United Kingdom	Telemanipulation	Operators must have a comprehensive engineering understanding of the systems capabilities and limitations Understand the system, but not the engineering behind it	Healthcare (surgery), nuclear reactor maintenance, underwater exploration, ordnance disposal, space
Telemental Health and Artificial Intelligence: Knowledge and Attitudes of Saudi Arabian Individuals Toward AI-Integrated Telemental Health	Alhur et al. (2023)	Journal of Population Therapeutics and Clinical Pharmacology	Saudi Arabia	Telemental health	Trust in protection of personal information, trust in privacy protection, acceptance of telemental health services	Mental health
Patients’ Trust in Artificial Intelligence-based Decision-making for Localized Prostate Cancer: Results from a Prospective Trial	Rodler et al. (2024)	European Urology Focus	Germany	AI in clinical workflows	Affinity for technology is associated with trust in AI, higher trust in AI controlled by a physician than not by a physician	Healthcare (diagnostic and therapeutic AI applications)
American public opinion on artificial intelligence in healthcare	Rojahn et al. (2023)	PLoS One	USA	Medical AI	Perception and trust of AI in healthcare	Healthcare
What is the future of artificial intelligence in obstetrics? A qualitative study among healthcare professionals	Fischer et al. (2023)	BMJ Open	Netherlands	Medical AI	validation, explainability, successful personal experience	Healthcare (obstetrics)
Trust criteria for artificial intelligence in health: normative and epistemic considerations	Kostick-Quenet et al. (2024)	Journal of medical ethics	USA	Clinical decision support systems	epistemic trust, accuracy/validity, relational trust, personal belief-based trust	Healthcare
Going under Dr. Robot’s knife: the effects of robot anthropomorphism and mortality salience on attitudes toward autonomous robot surgeons	Sonmez (2024)	Psychology and health	Turkey	Autonomous robot surgeons	Trust in robot surgeons	Healthcare
Theory of trust and acceptance of artificial intelligence technology (TrAAIT): An instrument to assess clinician trust and acceptance of artificial intelligence	Stevens and Stetson (2023)	Journal of Biomedical Informatics	USA	AI for clinical workflow	Explains clinician trust in AI; perception of AI trustworthiness (information credibility, system performance, application value)	Healthcare
Physician views of artificial intelligence in otolaryngology and rhinology: A mixed methods study	Asokan et al. (2023)	Laryngoscope Investigative Otolaryngology	USA	Clinical AI applications	Interviewees consistently valued two characteris-tics that trustworthy AI would have: agreement with their own clinicaljudgment and with expert analysis	Healthcare (otolaryngology and rhinology)
Modeling the influence of attitudes, trust, and beliefs on endoscopists’ acceptance of artificial intelligence applications in medical practice	Schulz et al. (2023)	Frontiers in public health	Singapore	Clinical AI applications	Trust attitudes impacting AI acceptance, trustwortiness, usefulness, familiarity	Healthcare
What if your patient switches from Dr. Google to Dr. ChatGPT? A vignette-based survey of the trustworthiness, value, and danger of ChatGPT-generated responses to health questions.	Van Bulck and Moons (2024)	European Journal of Cardiovascular Nursing	Belgium	ChatGPT	Nuanced and comprehensive responses increase trust, missing information, wording and tone	Healthcare
Exploring the feasibility of an artificial intelligence based clinical decision support system for cutaneous melanoma detection in primary care – a mixed method study.	Helenason et al. (2024)	Scandinavian Journal of Primary Health Care	Sweden	Clinical decision support systems	Diagnostic accuracy, importance of clinician trust, certified in the field, recommended by dermatologists	Healthcare (primary care)
Trust and stakeholder perspectives on the implementation of AI tools in clinical radiology	Bergquist et al. (2024)	European Radiology	Sweden	Decision support systems	Reliability, transparency, quality verification, interorganizational compatibility	Healthcare (radiology)
AI-Driven Transformations in Healthcare Marketing: A Qualitative Inquiry Into the Evolution and Impact of Artificial Intelligence on Online Strategies	Shahzad Khan et al. (2024)	Journal of Population Therapeutics and Clinical Pharmacology	Pakistan	AI in healthcare online marketing	maintaining privacy and data security; data usage transparency; clear communication about applications; respecting patients autonomy	Healthcare (marketing)
Robots for surgeons? Surgeons for robots? Exploring the acceptance of robotic surgery in the light of attitudes and trust in robots	Szabo et al. (2024)	BMC psychology	Hungary	Robotic surgery	Level of interaction, predictability, reliability, errors, reputation of the designer, appearance, willingness to trust, mental modeling, trus experience, cultural influence, sense of competence, perceived stress, received risk	Healthcare
Women’s perceptions and attitudes toward the use of AI in mammography in Sweden: a qualitative interview study	Johansson, et al. (2024)	BMJ Open	Sweden	AI in mammography	Trust in healthcare system dictates trust in AI; patient trust	Healthcare (mammography)
Perceptions of Artificial Intelligence Use in Primary Care: A Qualitative Study with Providers and Staff of Ontario Community Health Centers	Nash et al. (2023)	Journal of the American Board of Family Medicine	Canada	AI in primary care	Confidence, accuracy, prior negative experiences	Healthcare (primary care)
Supporting mental health self-care discovery through a chatbot	Moilanen et al. (2023)	Frontiers in Digital Health	Finland	Conversational agent	Security and integrity of systems	Mental health
Impact of cognitive workload and situation awareness on clinicians’ willingness to use an artificial intelligence system in clinical practice	Choudhury and Asan (2023)	IISE Transactions on Healthcare Systems Engineering	USA	Clinical decision support system	Situation awareness, effect of riskk perception on trust in AI, increasing cognitive workload decreases trust in AI	Healthcare
Investigating the Impact of User Trust on the Adoption and Use of ChatGPT: Survey Analysis	Choudhury and Shamszare (2023)	Journal of Medical Internet Research	USA	ChatGPT	Trust influences use; centrality of trust in technology adoption; relationship between trust and use	Healthcare
The (Im)perfect Automation Schema: Who Is Trusted More, Automated or Human Decision Support?	Rieger et al. (2024)	Human factors	Germany	AI support agents assisting with X-ray	Trust attitude and trust behaviors	Healthcare, decision support systems broadly
The effect of machine learning explanations on user trust for automated diagnosis of COVID-19	Goel et al. (2022)	Computers in Biology and Medicine	Australia	Deep learning	Trust of clinicians for complex decision tasks	Medical diagnostics
Promoting Healthcare Workers’ Adoption Intention of Artificial-Intelligence-Assisted Diagnosis and Treatment: The Chain Mediation of Social Influence and Human-Computer Trust	Cheng et al. (2022)	International Journal of Environmental Research and Public Health	China	AI-assisted diagnosis and treatment	Human-computer trust	Healthcare (dentistry)
“I do not think people are ready to trust these algorithms at face value”: trust and the use of machine learning algorithms in the diagnosis of rare disease	Hallowell et al. (2022)	BMC medical ethics	United Kingdom	AI for clinical decision making	Trustworthiness	Healthcare/computational phenotyping
Effect of risk, expectancy, and trust on clinicians’ intent to use an artificial intelligence system - Blood Utilization Calculator	Choudhury et al. (2022)	Applied Ergonomics	USA	AI-based decision support system	Expectancy, trust, and perceptions	Healthcare
Does AI explainability affect physicians’ intention to use AI?	Liu et al. (2022)	International Journal of Medical Informatics	Taiwan	Clinical support explainable AI	Technology trust, perceived value, explainability,	Healthcare
Patients’ Perspectives on Artificial Intelligence in Dentistry: A Controlled Study	Kosan et al. (2022)	Journal of Clinical Medicine	Germany	AI-based diagnosis	Intentionality, honesty, benevolence, neuroticism, reliability, functionality	Dentistry
Hospital-wide survey of clinical experience with artificial intelligence applied to daily chest radiographs	Shin et al. (2023)	PLoS ONE	South Korea	AI-based lesion detection software	Subjective trust levels	Healthcare/radiology
What if you have a humanoid AI robot doctor?: An investigation of public trust in South Korea	Kim and Kim (2022)	Journal of communication in healthcare	USA	Humanoid robots	Communication, gender	Healthcare
The roles of trust, personalization, loss of privacy, and anthropomorphism in public acceptance of smart healthcare services.	Liu and Tao (2022)	Computers in Human Behavior	China	AI-based smart healthcare services	Perceived: usefulness & ease of use, personalization, loss of privacy, anthropomorphism, gender, age, usage experience	Healthcare
Interacting with medical artificial intelligence: Integrating self-responsibility attribution, human–computer trust, and personality.	Huo et al. (2022)	Computers in Human Behavior	China	AI broadly	Self-responsibility attribution, agreeableness, conscientiousness, Big 5 personality traits,	Healthcare
Associations between literacy and attitudes toward artificial intelligence–assisted medical consultations: The mediating role of perceived distrust and efficiency of artificial intelligence.	Yi-No Kang et al. (2023)	Computers in Human Behavior	Taiwan	AI-assisted medical consultations	Health literacy, digital literacy, perceived distrust, and efficiency of AI	Healthcare
Do People Favor Artificial Intelligence Over Physicians? A Survey Among the General Population and Their View on Artificial Intelligence in Medicine	Yakar et al. (2022)	Value in Health	Netherlands	AI broadly	General attitude toward AI, distrust and accountability	Healthcare
Explainability does not improve biochemistry staff trust in artificial intelligence-based decision support	Lancaster Farrell (2022)	Annals of Clinical Biochemistry	Australia	AI decision support	Explainability	Healthcare
Artificial intelligence and the future of midwifery: What do midwives think about artificial intelligence? A qualitative study	Çitil and Çitil Canbay (2022)	Health care for women international	Turkey	AI broadly	Expectations included the advantages and conditional acceptance of robotic technology, prejudices reflected perceived shortcom-ings, lack of human competencies, and trust issues.	Healthcare/midwifery
Clinicians’ Perceptions of Artificial Intelligence: Focus on Workload, Risk, Trust, Clinical Decision Making, and Clinical Integration.	Shamszare and Choudhury (2023)	Healthcare	USA	AI broadly	Perception of AI-induced workload, AI risk, trust in AI, and AI-basedclinical decision making.	Healthcare
Determinants of patient trust in gastroenterology televisits: Results of machine learning analysis: Determinants of Patient Trust in Televisits	Costantino et al. (2022)	Informatics in Medicine Unlocked	Italy	AI broadly	Trust in the medical center, trust in the treatment, employment of a user-friendly video service, and data protection policies.	Healthcare
UK reporting radiographers’ perceptions of AI in radiographic image interpretation - Current perspectives and future developments	Rainey et al. (2022)	Radiography	Ireland	AI reporting	Trustworthiness, explainability, interpretability	Healthcare/radiology
A mixed-methods feasibility study of a novel AI-enabled, web-based, clinical decision support system for the treatment of major depression in adults	Qassim et al. (2023)	Journal of Affective Disorders Reports	Canada	Clinical decision support systems	Understanding, feasability, trust	Healthcare
Determinants of physicians’ intention to use AI-assisted diagnosis: An integrated readiness perspective.	Hsieh (2023)	Computers in Human Behavior	Taiwan	AI in medical imaging	Social value, emotional value, epistemic value, mistrust	Healthcare

### Definitions of trust

Of the 40 included articles, 15 articles explicitly defined trust, 10 of the articles defined trust in relation to technology, three of the articles took a more general, psychological approach to defining trust and two of the articles defined trust in the context of the healthcare system (Table 3). Although there were similarities among the definitions used, no two articles included in our study used the same trust definition. The differing definitions of trust are showcased in Table 4.

**Table 3:** Summary description of included studies.

Study characteristic	Number of studies
Methodology	
Quantitative	20
Qualitative	12
Mixed-methods	8
Type of healthcare	
Primary care	4
Surgery (including robotic surgery)	4
Mental health	2
Dentistry	2
Obstetrics and midwifery	2
Hematology	2
Cardiology	1
Otolaryngology	1
Gastroenterology	1
Radiology	1
Dermatology	1
Healthcare marketing	1

**Table 4:** Breakdown of the type of trust defined in the included studies.

Type of trust	Number of studies
Definitions of trust	15
Trust in technology	10
Trust (psychology)	3
Trust in healthcare	2

### Results

An inductive approach to thematic analysis was employed, allowing themes to emerge directly from the data without being shaped by pre-existing theoretical frameworks. Thematic analysis of the included articles revealed 3 main themes relating to the factors affecting user trust of AI in healthcare. These themes emerged as *(1) AI Literacy, (2) AI Psychology,* and *(3) AI Utility.* Each theme was made up of different subthemes. Table 5 shows a breakdown of the number of articles placed in each theme and subtheme. Many articles discuss more than one theme or subtheme, and are included in multiple sections (Table 6).

**Table 5:** Definitions of trust from included articles where trust was explicitly defined.

Technology definitions	Psychology definitions	Healthcare definitions
“Willingness to rely on the system, based on confidence that it will behave as expected” (Louca et al., 2023, p. 2)	“Trust depends on the interaction between the involved parties and should be understood as an ongoing process of establishing faith to reduce complexity. The social context is important for the interplay between a trustor and a trustee and consists of activities and strategies that will increase confidence between the involved parties. Human actors come to trust each other or an AI system because of the role a trustee plays in the larger system, such as the organization” (Bergquist et al., 2024, p. 339)	“A psychological mechanism driving clinician’s adoption behaviors in clinical environments in medical contexts” (Schulz et al., 2023, p. 2)
“The need to meet expectations and to be able to rely on automated systems to achieve a goal” (Szabo et al., 2024, p. 2)	“Trust is a relational concept—a disposition or intentional attitude—which is associated with situations of uncertainty, relations of dependency and expectations about future behavior/intentions” (Hallowell et al., 2022, p. 1)	“Patient trust in a telemedicine service can be considered as the patient’s willingness to rely on that health service (and the factors that make up that service) as a part of their treatment. Trust is a patient’s willingness to rely on a telemedicine service for personal gain (such as improved quality of care, time savings, or avoiding attendance at hospital during the COVID-19 pandemic). It has been demonstrated that trust is multidimensional and is most likely the sum of trust in several factors that constitute the telemedicine service (care organization, professional staff, treatment, technology), each of which can be trusted to a greater or lesser extent” (Costantino et al., 2022, p. 2)
“The attitude that an agent will help achieve an individual’s goals in a situation characterized by uncertainty and vulnerability” (Moilanen et al., 2023, p. 3)	“Defined trust as the willingness to bear the impact of others’ behavior on individuals, even if the individuals are not able to monitor or control the consequence of others’ behavior. In social psychology, trust comes from one’s perceived credibility and benevolence for an item or a person” (Liu et al., 2022, p. 2)	
“A user’s willingness to take chances based on the recommendations made by technology. This implies that the user believes that the technology has the capacity to execute a particular task accurately while keeping in mind the possibility of negative outcomes” (Choudhury and Shamszare, 2023, p. 3)		
“Willingness to accept a computer-generated recommendation” is an observable sign of user trust” (Goel et al., 2022, p. 3)		
“HCT (human computer trust) is the degree to which people have confidence in AI systems and are willing to take action. Trust is considered an attitude intention, which could directly influence acceptance and help people make cognitive judgments by decreasing risk perception and enhancing benefit perception. HCT is an attitude of trust that stems from the interaction between human and AI” (Cheng et al., 2022, p. 5)		
“The willingness to make oneself vulnerable to actions taken by the trusted party based on the feeling of confidence or assurance” (Liu et al., 2022, p. 2)		
“An individual’s willingness to be vulnerable to a technology based on one’s expectations of the technology’s predictability, reliability, and utility” (Liu et al., 2022, p. 2)		
“Trust serves as a central mechanism in explaining the relationship between individuals’ beliefs about technology characteristics and their acceptance behavior” (Liu and Tao, 2022, p. 2)		
“HCT (human-computer trust) is defined as an attitude of trust that arises during the interaction between human and AI” (Huo et al., 2022, p. 3)		
“Mistrust in AI-assisted tools refers to users’ perception that predictive models of AI-assisted tools are not trustworthy” (Hsieh, 2023, p. 4)		

**Table 6:** Breakdown of identified categories and subcategories.

Primary factors of trust	Number of studies
AI literacy	32
Trust enough to use	20
Understanding of AI systems	13
Explainability	10
AI psychology	27
More likely to trust	16
Understanding of trust	1
Human validation of AI	16
Distrust of AI	3
Trust repair	5
AI utility	26
Anthropomorphism	4
Trust/privacy/protection	9
Perceived value	19

#### Theme 1: AI Literacy for acceptance, understanding, and explainability

A major theme in the literature is centered around the need for user understanding of the input, processes and outputs of the AI system. 32 articles referred to “user literacy” as being a factor affecting either patient or physician trust in AI. The need for literacy was further broken down in the literature in terms of *acceptance*, *understanding*, and *explainability*.

##### Acceptance–trust enough to use

20 articles discussed the need for a level of trust that was high enough to influence the adoption and acceptance of AI systems into the healthcare system (Alhur et al., 2023; Bergquist et al., 2024; Cheng et al., 2022; Choudhury et al., 2022; Choudhury and Shamszare, 2023; Çitil and Çitil Canbay, 2022; Costantino et al., 2022; Hsieh, 2023; Kim and Kim, 2022; Kosan et al., 2022; Kostick-Quenet et al., 2024; Liu and Tao, 2022; Nash et al., 2023; Rieger et al., 2024; Shin et al., 2023; Sonmez, 2024; Stevens and Stetson, 2023; Szabo et al., 2024; Viberg Johansson et al., 2024). Many articles stressed the importance of system accuracy, reliability, and overall trustworthiness as major factors in building enough trust in a system to adopt it into practice (Bergquist et al., 2024; Fischer et al., 2023; Hsieh, 2023; Kostick-Quenet et al., 2024; Nash et al., 2023; Rieger et al., 2024; Stevens and Stetson, 2023; Shin et al., 2023). The level of risk associated with the task given to the AI system can also influence physician trust in the system. For example, in a study focusing on a clinical decision support system for survival rate in cardiology patients, Kostick-Quenet et al. (2024) state that: “some physicians noted that they demand higher levels of accuracy for estimates intended to inform choices about pursuing an intervention with life-or-death implications versus those intended to predict postoperative adverse events and manage postoperative care”. Physicians want to trust that the system will work as expected, and patients want to trust that their physician trusts the system will work as expected (Cheng et al., 2022; Choudhury et al., 2022). Two studies noted that many physicians who were initially apprehensive toward the implementation of AI into healthcare gained trust in the system after working with the system (Hsieh, 2023; Shin et al., 2023).

##### Understanding–knowledge of AI systems

Understanding the inner workings of AI system operation was discussed in 13 articles (Asokan et al., 2023; Bergquist et al., 2024; Choudhury and Shamszare, 2023; Hallowell et al., 2022; Hsieh, 2023; Shahzad Khan et al., 2024; Kostick-Quenet et al., 2024; Liu et al., 2022; Louca et al., 2023; Moilanen et al., 2023; Nash et al., 2023; Qassim et al., 2023; Viberg Johansson et al., 2024). Many articles iterated that in using AI systems, physicians do not require a comprehensive engineering understanding of how the system works but would rather have transparency in terms of the datasets used for building the algorithms, and on the development and testing processes (Asokan et al., 2023; Bergquist et al., 2024; Choudhury and Shamszare, 2023; Hallowell et al., 2022; Hsieh, 2023; Shahzad Khan et al., 2024; Kostick-Quenet et al., 2024; Louca et al., 2023; Moilanen et al., 2023; Qassim et al., 2023). This point is illustrated by Kostick-Quenet et al. (2024), who state that, “both patients and physicians from our study emphasized a desire to know more about the nature of data sets—rather than algorithms—used to train algorithmic models, in order to gage relevance of outputs for making inferences about target users or subjects.” According to Qassim et al., physicians view AI systems as a supplement to their clinical judgment; therefore, they do not require a system understanding (Qassim et al., 2023). Three studies found that transparency on the limitations of the system was key to developing physician trust (Moilanen et al., 2023; Liu et al., 2022; Qassim et al., 2023). Asokan et al. (2023) highlight the need for transparency in the development and testing of AI systems, when they state that “respondents consistently desired transparency on how an AI tool was developed and tested.” In contrast, two studies did note that providing information on the algorithmic processes and inner workings of the AI systems did improve physician trust and intent to use (Choudhury and Shamszare, 2023; Hallowell et al., 2022). For example, Hallowell et al. (2022) state that: “trust must be built upon transparency about, and awareness of, how algorithms work, rather than having ‘blind faith’ in algorithmic output”.

##### Explainability–knowledge of AI outputs

Ten studies discuss how user trust in the AI system is built when the system can explain its final decision (Bergquist et al., 2024; Fischer et al., 2023; Goel et al., 2022; Helenason et al., 2024; Kostick-Quenet et al., 2024; Lancaster Farrell, 2022; Liu et al., 2022; Qassim et al., 2023; Rainey et al., 2022; Van Bulck and Moons, 2024). Many of these studies noted that physician trust in AI systems, most specifically in clinical decision support systems (CDSS), increases with system explainability (Goel et al., 2022; Lancaster Farrell, 2022; Liu et al., 2022; Qassim et al., 2023; Rainey et al., 2022). When analyzing the implementation of a CDSS for psychiatric use, Qassim et al. (2023) found that “doctors felt comfortable using the app in practice because they were, as per one physician, “never surprised” by the CDSS’s recommendations and the “AI explained reasoning behind its choices.” Two studies found that explanations become even more imperative when an AI response may differ from a clinician’s point of view and can aid in exploring different clinical treatments (Bergquist et al., 2024; Kostick-Quenet et al., 2024). The bias many radiologists tend to face is explained by Bergquist et al. (2024), when they state that “more recent cases tended to influence them [radiologists] the most, whereas the AI considered all cases it had been trained on and thus provided them with a more extensive frame of reference.”

#### Theme: 2: AI psychology and human development

The second theme, identified in 27 articles, explores the psychological factors underlying the development of trust in AI systems. This theme focused more on the population demographic and profile of individuals trusting the AI system. This theme was further broken down in the literature in terms of *human development*, *human motivation*, and *human relations*.

##### Human development—who is more likely to trust

In 16 articles, the literature pointed to individual traits or lived experiences that may make an individual more or less likely to trust an AI system (Asokan et al., 2023; Choudhury and Asan, 2023; Hsieh, 2023; Huo et al., 2022; Kim and Kim, 2022; Kosan et al., 2022; Liu and Tao, 2022; Moilanen et al., 2023; Nash et al., 2023; Schulz et al., 2023; Sonmez, 2024; Stevens and Stetson, 2023; Szabo et al., 2024; Yakar et al., 2022; Yi-No Kang et al., 2023). Three studies stated that experience using AI systems is a precursor to increased trust in the system (Asokan et al., 2023; Liu and Tao, 2022; Schulz et al., 2023). Schulz et al. (2023) highlight the need for increased AI exposure when they state: “our findings show that trust and beliefs need to go hand in hand with exposure to AI. Therefore, introductions to AI need to be more effectively done through trust enhancers, such as involving trusted professional sources.” Two studies found that positive experiences with AI systems lead to increased trust (Moilanen et al., 2023; Szabo et al., 2024), whereas negative experiences may lead to decreased trust in the systems (Moilanen et al., 2023; Nash et al., 2023). Despite most of the literature agreeing that experience with AI technologies increased physician trust and willingness to implement new technologies, one of the included studies did not find any significant correlation between trust and previous experience (Choudhury and Asan, 2023). This is highlighted in the study by Choudhury et al. (2022), on the implementation of blood utilization calculators (BUC) into clinical practice when they state that “according to our study, clinicians’ experience of using the AI system (BUC) or their familiarity with AI technology, in general, had no significant impact on their trust in–or intent to use–BUC.” Four studies noted that men tend to show increased levels of trust toward AI systems in the healthcare field rather than women (Çitil and Çitil Canbay, 2022; Kim and Kim, 2022; Liu and Tao, 2022; Sonmez, 2024). Conversely, Hsieh (2023) found no significant effect of gender on AI usage. Age was discussed as a factor affecting trust in AI systems in three studies (Kosan et al., 2022; Liu and Tao, 2022; Stevens and Stetson, 2023). Stevens and Stetson (2023) note the effect of age when they state: “as the clinician’s age group increased, the trustworthiness of AI became increasingly important to their willingness to use it.” Another study emphasized that elderly patients tend to be more skeptical of technology being introduced into their healthcare, and that, in general, younger individuals are more trusting of technology (Kosan et al., 2022). Two studies noted that higher levels of education often correlated with higher levels of technology trust (Kosan et al., 2022; Yi-No Kang et al., 2023). Notably, higher education can increase health literacy among patients, and an increased health literacy has a positive effect on trust of AI systems in the healthcare field (Yi-No Kang et al., 2023). One study found that individuals who have a higher cumulative affinity for technology tend to have more trust in AI being integrated into the healthcare field (Rodler et al., 2024).

##### Human motivation–experience of trust

When analyzing the factors that influence trust in AI, one article in our review highlighted certain psychological aspects of trust that, while not specific to AI, are fundamental to the development of trust in any context (Hallowell et al., 2022). Hallowell et al. (2022) explain that if the “cost” of not trusting something is greater than the “cost” of trusting, then individuals may act as though they have trust in a particular thing, but that “this does not indicate the existence of a fully-fledged trust relationship.” They also stress the idea that “developing trust in any technology is reliant on one’s experience of using it; trust is learnt” (Hallowell et al., 2022).

##### Human relations–human validation of AI

Sixteen articles included in our review pointed to the idea that trust in the decisions made by AI systems is built when a human or professional in the field validates the output given by an AI system (Asokan et al., 2023; Cheng et al., 2022; Fischer et al., 2023; Helenason et al., 2024; Hallowell et al., 2022; Kosan et al., 2022; Kostick-Quenet et al., 2024; Nash et al., 2023; Qassim et al., 2023; Rodler et al., 2024; Rojahn et al., 2023; Schulz et al., 2023; Shin et al., 2023; Sonmez, 2024; Szabo et al., 2024; Van Bulck and Moons, 2024; Viberg Johansson et al., 2024). From the point of view of the patient, one study found that generally, “the public significantly prefers a human physician over an AI system” (Rojahn et al., 2023). However, three studies found that when AI is endorsed by their clinical team, patients are more likely to trust the AI system (Hallowell et al., 2022; Kostick-Quenet et al., 2024; Qassim et al., 2023). Additionally, it was found that patients have more trust in AI systems controlled by physicians than those not controlled by a physician (Hallowell et al., 2022; Qassim et al., 2023; Rodler et al., 2024). This is highlighted by Hallowell et al. (2022) who found: “as far as our interviewees were concerned if a trusted person—your doctor—uses AI, then you are more likely to trust the algorithmic output.” Eight studies found that physicians are more likely to trust the system when there is proof of validation by professionals within their field (Asokan et al., 2023; Fischer et al., 2023; Hallowell et al., 2022; Helenason et al., 2024; Kostick-Quenet et al., 2024; Nash et al., 2023; Qassim et al., 2023; Schulz et al., 2023). Kostick-Quenet et al. (2024) highlight the need for human validation by saying: “both physicians and patients explained that they would be more likely to trust algorithmic estimates that were endorsed by members of the medical community who are themselves perceived as reputable and trustworthy.” Five studies report physicians have improved trust in the AI system when it matches their clinical judgment (Asokan et al., 2023; Hallowell et al., 2022; Helenason et al., 2024; Qassim et al., 2023; Viberg Johansson et al., 2024), as described in the study by Qassim et al. (2023) when they state that “5/7 doctors reported they trusted the tool because, as one doctor put it, it “was aligned with doctor’s clinical opinions and was good reinforcement from an exogenous source.”

##### Human attitudes–distrust of AI

An aspect of AI Psychology captured in three of the included articles is the idea of distrust, which captures those who are apprehensive toward the implementation of AI systems in the healthcare field (Hsieh, 2023; Van Bulck and Moons, 2024; Yi-No Kang et al., 2023). One study states that mistrust of the systems is a significant barrier to physician implementation, and states a lack of transparency and accuracy as major factors influencing this mistrust (Hsieh, 2023). One study reports that when asked about patients using ChatGPT to seek medical advice, many experts were worried about the trustworthiness of the responses given, noting that “certain information was missing, too vague, a bit misleading, and not written in a patient-centered way” (Van Bulck and Moons, 2024). Another study found that “individuals with higher levels of digital literacy have poor attitudes toward AI-assisted medical consultations because of their higher perceived distrust of AI” (Yi-No Kang et al., 2023).

##### Human growth–trust repair

Five articles in our review explore the idea of trust in an AI system, specifically after the AI system has made an error (Huo et al., 2022; Nash et al., 2023; Rieger et al., 2024; Rojahn et al., 2023; Viberg Johansson et al., 2024). One article expressed that in general, patients have much greater trust in human physicians than in AI systems, even when made aware of the greater likelihood of a human making a biased error (Rojahn et al., 2023). Two studies found that patients feel as though a human can learn from past mistakes, but that AI systems cannot grow in the same capacity, making trust repair more difficult in human-machine relationships (Rojahn et al., 2023; Viberg Johansson et al., 2024). This idea is contrasted by research conducted by Rieger et al. (2024), which found no differences in forgiveness and trust restoration after failure between an AI decision support agent and a human. From the point of view of the physician, one study reports that previous negative experiences with technology implementation can deter physicians from trusting new AI technologies (Nash et al., 2023).

#### Theme: 3: AI utility

The final theme identified in this review was present in 26 articles and focused on the utility of the AI system and how this impacts user trust. We have further broken this main theme into the categories of *anthropomorphism*, *privacy and protection*, and *perceived value*.

##### Anthropomorphism

The literature defines anthropomorphism as “the pervasive human tendency to attribute human characteristics to non-human entities” (Sonmez, 2024). There were four articles that discussed the idea of anthropomorphic AI systems, and found the literature is in consensus that patient trust in AI systems, such as surgical robots, chatbots or robot doctors, is improved when these machines exemplify human-like traits (Sonmez, 2024; Kim and Kim, 2022; Liu and Tao, 2022; Moilanen et al., 2023). Moilanen et al. (2023) highlight this in their study regarding chatbot use in mental healthcare when they state that “chatbot behavior and human likeness are essential factors informing trust in chatbots.” Two studies note that increased trust in anthropomorphic AI systems stems from the idea of a social presence (Kim and Kim, 2022; Liu and Tao, 2022). Liu and Tao (2022) describe this in their study when they state that:

“We found that while anthropomorphism failed to produce direct effect on behavioral intention, it indeed exerted an indirect effect on behavioral intention through the mediating role of trust. It is likely that, when people are interacting with anthropomorphic smart healthcare services, the feeling of trust would be emerged due to the perception of a social presence”.

They additionally noted that anthropomorphism is increasingly important for developing trust in females and younger adults (Liu and Tao, 2022).

##### Privacy and protection

Nine articles in our study discuss the interrelationship between trust and the protection of privacy and data (Alhur et al., 2023; Asokan et al., 2023; Bergquist et al., 2024; Costantino et al., 2022; Shahzad Khan et al., 2024; Liu and Tao, 2022; Moilanen et al., 2023; Nash et al., 2023; Viberg Johansson et al., 2024). Four studies emphasize that inadequate protection of patient privacy is a major patient concern regarding the implementation of AI into the healthcare system and can have significant effects on patient trust in these systems being incorporated into their care (Asokan et al., 2023; Moilanen et al., 2023; Nash et al., 2023; Yi-No Kang et al., 2023). These privacy concerns are highlighted in the study conducted by Liu and Tao (2022), looking at public acceptance of smart healthcare services, when they state that “loss of privacy did not directly influence behavioral intention, but it was found to have a negative influence on trust, indicating that consumers concerned with privacy are less likely to trust such services.” One study found these concerns become increasingly relevant in sensitive areas of healthcare where data privacy is much more important, such as mental health (Moilanen et al., 2023). One study found that trust increases when patient privacy concerns are addressed and robust security measures are put in place (Alhur et al., 2023). Furthermore, two studies found that patient trust in AI systems is built when privacy and data security are maintained (Costantino et al., 2022; Shahzad Khan et al., 2024). Bergquist et al. (2024) found that from the physician’s point of view, having control over the data is an important factor in determining trust.

##### Perceived value

19 articles included in our study found that the perceived value of the AI tool was a factor influencing trust (Asokan et al., 2023; Bergquist et al., 2024; Cheng et al., 2022; Choudhury et al., 2022; Çitil and Çitil Canbay, 2022; Costantino et al., 2022; Helenason et al., 2024; Hsieh, 2023; Liu et al., 2022; Liu and Tao, 2022; Nash et al., 2023; Qassim et al., 2023; Rojahn et al., 2023; Schulz et al., 2023; Shamszare and Choudhury, 2023; Shin et al., 2023; Stevens and Stetson, 2023; Szabo et al., 2024; Yakar et al., 2022; Yi-No Kang et al., 2023). Without added value to the patient-physician encounter or to clinical workflow, the adoption of AI systems would not be effective, as identified by Liu et al., when they state, “if medical AI is of little value to physicians, physicians will resist accepting AI” (Liu et al., 2022). Seven studies report that among both patients and physicians, trust and willingness to adopt a particular AI system increased when the user perceived the system to be useful (Alhur et al., 2023; Cheng et al., 2022; Liu and Tao, 2022; Qassim et al., 2023; Schulz et al., 2023; Stevens and Stetson, 2023; Yi-No Kang et al., 2023). Additionally, nine studies found that for both patients and physicians, ease of use of the system had major implications on trust and willingness to use. Three studies report that patients felt that the AI system needed to be convenient for them to use and add efficiency to their encounters (Alhur et al., 2023; Yakar et al., 2022; Yi-No Kang et al., 2023). Regarding physicians, one study that focused on otolaryngologists’ views on AI noted that “physicians valued efficiency more than accuracy or understanding AI algorithms and design” (Asokan et al., 2023). Six studies noted that to trust a system enough to implement it into clinical practice, it must be capable of speeding up complex tasks, reducing reading times or reducing physician workload (Choudhury et al., 2022; Çitil and Çitil Canbay, 2022; Hsieh, 2023; Qassim et al., 2023; Shamszare and Choudhury, 2023; Shin et al., 2023). The relationship between physician workload and trust in AI is highlighted by Shamszare and Choudhury (2023) when they state that “clinicians who view AI as a workload reducer are more inclined to trust it and are more likely to use it in clinical decision making.” To add to efficiency and ease of use, two studies found that physicians require that AI systems be compatible with other systems and practices within their organization (Bergquist et al., 2024; Viberg Johansson et al., 2024). This idea is highlighted by the study investigating the implementation of AI tools in radiology by Bergquist et al. (2024), as their results state that:

“Radiologists’ trust in AI depends on the experience that AI is compatible with other systems and practices in the organization, increasing their capacity and providing control. Trust in AI emerges when a variegated range of data formats are integrated into existing modalities so that experts across organizational or functional boundaries can share and use data to collaborate efficiently and safely”

Finally, three studies found that physicians must believe that utilizing an AI system will lower their risk of missed diagnoses and will be an added aid to confirm their diagnoses (Hsieh, 2023; Qassim et al., 2023; Shin et al., 2023).

## Discussion

AI holds significant potential to enhance decision-making, increase efficiency, and transform existing healthcare practices. Research indicates that the impact of automated systems will depend on whether and how human factors are incorporated into their design (Asan et al., 2020). Thus, as AI continues to be implemented throughout the healthcare system, considering the factors that impact patient and physician trust in AI technologies is of vital importance (Baduge et al., 2022; Glikson and Woolley, 2020). Trust is a complex construct involving many factors, and it plays a critical role in both human relationships and human-machine interactions (Asan et al., 2020). Our review identified that only 35% of the included studies provided an explicit definition of trust. Furthermore, none of the articles utilized the same definition, underscoring the complexity of evaluating this variable using a standardized approach. Definitions of trust were subsequently categorized according to the context under evaluation, including trust in technology, psychological perspectives on trust, and trust within healthcare settings. Across various disciplines, workers’ trust in AI technology is an important aspect of the successful integration of AI into a workplace (Glikson and Woolley, 2020). This rapid review addresses the factors that contribute to developing a trusting relationship in AI technologies within the healthcare industry.

Several factors were identified as essential prerequisites for fostering trust in AI technologies, highlighting the minimum requirements that AI systems must meet to gain user confidence. These factors include AI technologies addressing privacy concerns, maintaining accuracy, providing credible information, high levels of system performance, addressing biases, and overall reliability (Bergquist et al., 2024; Fischer et al., 2023; Kostick-Quenet et al., 2024; Stevens and Stetson, 2023).

When users understood the capabilities and limitations of the AI system they were using, a trusting relationship could be developed (Louca et al., 2023; Moilanen et al., 2023; Viberg Johansson et al., 2024). Both patients and physicians expressed a desire to understand how the overall system was developed and how datasets are utilized to build and refine the algorithms used in healthcare settings (Asokan et al., 2023; Bergquist et al., 2024; Kostick-Quenet et al., 2024; Liu et al., 2022). Gender was linked to attitudes toward AI technology, with females having less trust and more negative attitudes toward technology compared to males in all but one study (Hsieh, 2023) where gender was insignificant in predicting physician trust (Çitil and Çitil Canbay, 2022; Hsieh, 2023; Kim and Kim, 2022; Liu and Tao, 2022; Sonmez, 2024). Sonmez (2024) specifically found that women had a higher risk aversion compared to men, with less positive attitudes, regardless of the human-likeness of AI. Additionally, experience using technology was a contributing factor leading to trust in AI technology. Those with more experience with technology were more likely to trust AI technology and their decisions (Asokan et al., 2023; Moilanen et al., 2023; Schulz et al., 2023). In contrast, previous negative experiences caused distrust and deterred the use of the systems (Nash et al., 2023). Although some studies report the impact of age on trust in technology, one study in this review found that neither prior experience nor age was a factor impacting trust in AI systems or intentions to utilize AI systems (Choudhury and Asan, 2023). This contradicts findings that demonstrated a significant impact of age on trust in AI technology, with older individuals generally exhibiting lower levels of trust in AI compared to younger individuals (Nash et al., 2023; Oksanen et al., 2020). Overall, the more familiar a user is with AI technology, the more likely they are to trust the system. Many studies have found explainable artificial intelligence to be an essential factor impacting user trust (Bergquist et al., 2024; Fischer et al., 2023; Goel et al., 2022; Helenason et al., 2024; Qassim et al., 2023). ^A^s outlined in Theme 1, several included studies reported higher adoption and comfort when AI provided interpretable outputs rather than “black box” predictions. This underscores explainability as a critical design feature for trustworthy AI in healthcare (Bergquist et al., 2024; Fischer et al., 2023; Goel et al., 2022; Helenason et al., 2024; Qassim et al., 2023). However, two studies found that explainability was not a factor that predicted whether physicians would or would not utilize AI technology and did not act as a factor to improve trust in healthcare contexts (Lancaster Farrell, 2022; Liu et al., 2022). Understanding the complexity of trust and how trust is built was another important factor influencing trust relationships. For example, trust relationships were impacted when users of AI technologies recognized that trust is a learned behavior and evaluated the overall impact of whether they were to trust or not trust a system (Hallowell et al., 2022).

Our review found that trust is built when the user believes there will be positive benefits from using the AI system. Positive perceptions regarding the usefulness of AI technologies resulted in greater user trust (Alhur et al., 2023; Stevens and Stetson, 2023). Specifically, physicians were likely to use and trust AI technologies if they found them to be efficient, easy to use, and useful in their practice (Asokan et al., 2023; Cheng et al., 2022; Hsieh, 2023; Liu and Tao, 2022; Nash et al., 2023; Qassim et al., 2023; Schulz et al., 2023; Shamszare and Choudhury, 2023; Yakar et al., 2022; Yi-No Kang et al., 2023). As the perceived value of an AI technology increases, physicians are more likely to rely on the system, thereby enhancing their trust in it (Bergquist et al., 2024; Goel et al., 2022; Liu et al., 2022). Furthermore, trust in the AI system was enhanced when the system could be seamlessly integrated into the physicians’ practice and demonstrated compatibility with existing systems (Bergquist et al., 2024).

The human-likeness of AI systems plays a key role in shaping patient trust, fostering a greater openness to incorporating AI technology into their care networks (Choudhury and Shamszare, 2023; Kim and Kim, 2022; Liu and Tao, 2022; Sonmez, 2024). The greater the degree of human-like attributes exhibited by AI technologies, such as surgical robots, the higher the likelihood that individuals will extend their trust in these systems, owing to an enhanced sense of social presence (Kim and Kim, 2022; Liu and Tao, 2022). Trust repair after system failure remains challenging for AI, though findings were mixed, as outlined in Theme 2 (Asokan et al., 2023; Moilanen et al., 2023). This reflects the psychological aspect of trust: human errors are often seen as part of a learning process, while AI mistakes are viewed as indicative of systemic flaws (Moilanen et al., 2023). Consequently, this underscores the necessity for effective trust repair strategies to rebuild confidence in AI systems within healthcare settings (Moilanen et al., 2023). This review found that different psychological and external influences can shape the feelings of the patient toward AI. Therefore, understanding these factors can help build patient acceptance of AI systems and build patient trust in AI even when it makes mistakes (Huo et al., 2022).

As detailed in Theme 2, trust in AI often follows a cascade: patients are more likely to trust systems endorsed by their physicians, and physicians are more comfortable when the technology has been validated by peers, experts, or reputable organizations. Rather than revisiting individual study findings, this reinforces the importance of human validation as a bridge between developers, clinicians, and patients (Asokan et al., 2023; Cheng et al., 2022; Fischer et al., 2023; Helenason et al., 2024; Hallowell et al., 2022; Kostick-Quenet et al., 2024; Qassim et al., 2023; Rodler et al., 2024; Sonmez, 2024). Trust was further enhanced if professionals outside the healthcare field, such as software engineers, program designers, and technology leaders, confirmed the trustworthiness of AI systems (Szabo et al., 2024). Physician trust would fluctuate, though, based on the result of the AI technology. For example, if the system results in decisions that the physician disagrees with, they may grow to distrust it (Hallowell et al., 2022). However, trust was enhanced if the system tended to come to the same decision as the physician (Asokan et al., 2023; Hallowell et al., 2022; Helenason et al., 2024; Qassim et al., 2023; Viberg Johansson et al., 2024). Additionally, physicians who recognize the potential for bias in the development of AI technologies tend to be more cautious and less likely to trust these systems sufficiently to integrate them into their clinical practice (Van Bulck and Moons, 2024). To trust a system, physicians want to be confident that the system is trained on a robust dataset and that these systems are constantly updating. This can lead to a disconnect between patients’ privacy of their data and accurately training the AI systems to be as accurate as possible (Bergquist et al., 2024). In a study conducted by Viberg Johansson et al. (2024) looking at women’s perceptions and attitudes toward the implementation of AI in mammography, patients were willing to share their data for life-saving measures or research purposes but were reluctant to share with private entities, as they considered it “an intrusion into their private lives.” Patients may not understand that data sharing with private entities is essential for technology advancements (Viberg Johansson et al., 2024).

The Organization for Economic Co-Operation and Development (OECD) states that trustworthy AI will be transparent and explainable, where the capabilities and limitations of the system are readily available and easily explained to users (OECD Legal Instruments, 2019). A major area of concern with AI in sensitive fields such as healthcare is the “black box” nature of the system. Output and decisions are often given without explanation behind them, making implementation and trust difficult (Liu et al., 2022). The introduction of explainable AI (XAI) refers to the actions and measures taken to ensure transparency in the AI system that is both explainable and interpretable (Adadi and Berrada, 2018; Liu et al., 2022). Implementing XAI could be a major step toward generating trust in AI systems and furthering their implementation. Trustworthy AI should have security and safety measures in place to avoid harm in the case of misuse or adverse conditions (OECD Legal Instruments, 2019). Finally, trustworthy AI should be accountable and ensure traceability of datasets, processes and decisions made by the system (OECD Legal Instruments, 2019). Because AI can be implemented in vastly different ways in different fields, major factors impacting trust in one field may vary greatly from those in another field. Understanding the key aspects that play a role in determining user trust in AI systems is important for the successful implementation of AI (Abeywickrama et al., 2023; Louca et al., 2023). The ability of the AI system to explain its decision-making process can reveal bias that a diagnosing practitioner may have been unaware of (Bergquist et al., 2024). Therefore, collaboration between technology programmers and healthcare professionals may ensure that the outputs of AI technology are valid and aligned with clinicians, resulting in stronger trust relationships (Nash et al., 2023). Furthermore, when patients believed the physician was making the final decision, not the AI system, they were more open to trusting AI technologies to assist with healthcare decisions (Rodler et al., 2024; Van Bulck and Moons, 2024; Viberg Johansson et al., 2024). However, studies found that patients preferred physicians over AI systems, even if trust was formed (Rojahn et al., 2023; Viberg Johansson et al., 2024). Thus, a cascading relationship exists in user trust for AI systems. For a patient to trust the system, the physician must first trust the system, and the physician’s trust depends on their confidence in the program developer who created it (Hallowell et al., 2022). From the patient’s perspective, a common barrier to trust in AI technologies is the concern over privacy invasion and data collection practices (Liu and Tao, 2022; Moilanen et al., 2023; Nash et al., 2023; Viberg Johansson et al., 2024). In contexts where users exhibit a moderate level of trust in AI systems, the implementation of direct safeguards for data protection and privacy significantly enhanced trust in these systems (Alhur et al., 2023). Healthcare organizations need to adopt standardized procedures for addressing data collection and management issues associated with AI technologies to foster trust among patients (Bergquist et al., 2024; Costantino et al., 2022). This includes physicians ensuring open communication and transparency with patients surrounding privacy and data security (Shahzad Khan et al., 2024).

To trust a system, physicians want to be confident that the system is trained on a robust dataset and that these systems are constantly updating. This can lead to a disconnect between patients privacy of their data and accurately training the AI systems to be as accurate as possible (Bergquist et al., 2024). Physicians may be more willing to share data for research purposes; however, patients may not understand that data sharing with private entities is essential for technology advancements (Viberg Johansson et al., 2024). Overall, the successful integration of AI technologies into healthcare will depend on fostering trust among both physicians and patients. The multifaceted nature of trust is evident, as well as the importance of transparency, system reliability, and privacy protections. As the development and implementation of AI evolves, understanding and addressing the factors that influence trust will be critical to ensure effective adoption and long-term success in improving healthcare outcomes.

## Limitations

This study has several limitations. Firstly, as this study is a rapid review, the search was less comprehensive than a scoping or systematic review. The scope of this study was limited to healthcare contexts, so we caution against any interpretations of findings beyond the healthcare setting. Future studies should take a broader approach to consider factors of trust in other contexts. Our search was limited to academic publications, so there could be valuable information in the gray literature that was excluded from this review.

## Future research

This review does not address methods for building trust between humans and artificial intelligence in healthcare settings; thus, future research should address this. Further studies should examine the factors of trust in AI outside of healthcare settings. Additionally, longitudinal studies should examine how trust evolves and explore effective recovery strategies related to AI errors. These studies could further investigate how initial impressions of AI evolve with sustained use. This may include investigating effective trust repair mechanisms, including system transparency, error communication strategies, and human oversight models, to restore confidence after AI failure. Future studies may adopt large-language models (LLMs) in order to assist with literature analysis. For the purposes of this review LLMs were not utilized as our goal was to ensure transparency, reproducibility, and methodological rigor by relying on traditional, human-driven review methods such as systematic searching, critical appraisal, and manual synthesis of findings. While LLMs have shown promise for assisting in scientific research and paper drafting, their capabilities, limitations, and best practices for reliable use in scholarly reviews were not yet well defined at the time of this work, and integrating them was considered outside the scope of our study.

An effort should be made to develop standardized metrics and validated scales to measure trust in AI systems consistently across healthcare contexts. The current literature varies and often involves non-comparable definitions of trust, making cross-study synthesis challenging. It also suggested that a co-design and participatory development framework is utilized, engaging clinicians, patients, and technology developers to ensure that AI tools align with user needs, workflows, and ethical expectations. Future studies should also investigate how trust is shaped across diverse cultural contexts and low-resource settings, as well as how trust develops within interdisciplinary teams that include not only clinicians but also information technology developers, administrators, and other stakeholders involved in AI implementation. Although this rapid review did not aim to evaluate or propose specific methodological frameworks, future work would benefit from the development and application of structured approaches—such as standardized trust metrics, longitudinal study designs to track trust over time, and co-design models that engage clinicians, patients, and developers—to guide the creation and assessment of trustworthy AI systems.

## Conclusion

Through a rapid review of the literature, our findings suggest that trust is imperative for the successful implementation of AI in healthcare settings and that there are numerous factors that contribute to patient and physician trust in AI systems. By examining broad categories that shape trust, including AI literacy, psychology and utility, this review underscores the complexity of building trust across diverse user groups. Trust in AI is not a static construct that is well defined but involves a dynamic interplay of system transparency, perceived value, and user experience. Furthermore, concepts such as anthropomorphism, privacy, and trust repair strategies must be considered when evaluating trust in AI systems. In order to use AI systems to their full potential in healthcare settings, developers, clinicians, and policymakers must collaborate to address these multifaceted aspects of trust. This involves designing AI systems that are transparent, explainable, and well-aligned with ethical standards to maintain robust privacy safeguards. Future research should continue exploring the nuanced relationships among trust factors and expand beyond healthcare to inform AI applications in other critical domains. Trustworthy AI systems not only have the ability to enhance clinical decision-making and efficiency but could pave the way for a more equitable and inclusive healthcare landscape.
